# A Rare Associated Anomaly in Tetralogy of Fallot: Scimitar Syndrome in an Infant

**DOI:** 10.21470/1678-9741-2020-0185

**Published:** 2021

**Authors:** Betül Çınar, Sezen Ugan Atik, Alper Güzeltaş

**Affiliations:** 1 Department of Pediatric Cardiology, Istanbul Mehmet Akif Ersoy Thoracic and Cardiovascular Surgery Center and Research Hospital, Istanbul, Turkey.

**Keywords:** Heart Atria, Vena Cava, Inferior, Scimitar Syndrome, Tetralogy of Fallot, Cyanosis

## Abstract

Pulmonary venous connections may be infrequently abnormal in patients with tetralogy of Fallot (TOF). A special subgroup of partial anomalous pulmonary venous return,"scimitar cyndrome", and its coexistence with TOF is less frequently reported. It may proceed unnoticed, as cyanosis already predominates in the clinical picture. This uncommon association must be kept in mind for patients with TOF who have an accessory flow in the inferior vena cava, especially when all pulmonary venous return to the left atrium is not clearly seen.

**Table:** 

Abbreviations, acronyms & symbols	
**TOF**	**= Tetralogy of Fallot**

## INTRODUCTION

Abnormal pulmonary venous connections, either total or partial, may accompany tetralogy of Fallot (TOF) in some patients, which is rarely reported in the literature^[^^1^^,^^2^^]^. It is even rarer to find a subgroup of partial pulmonary venous return anomaly in the form of scimitar syndrome in patients with TOF^[^^3^^]^. By definition, scimitar syndrome represents a partial anomalous pulmonary venous connection which is associated with systemic arterial supply from the abdominal aorta to some part of the lungs, along with concurrent lung hypoplasia. This anomalous pulmonary vein often drains into the inferior vena cava, nevertheless drainage into hepatic vein, right or left atrium or portal vein can occur.

Herein, we present an unusual coexistence of TOF with scimitar syndrome and our transcatheter+surgical treatment approach in a seven-month-old male infant.

## CASE REPORT

**Fig. 1:**
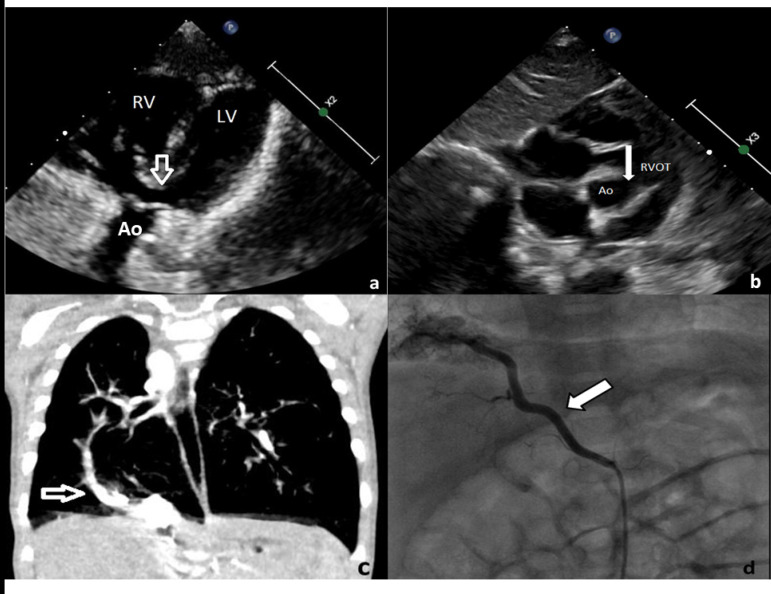
Echocardiographic 2D-modified subcostal view demonstrating (A) the subaortic VSD (white arrow), overriding aorta and right ventricular hyperthrophy, (B) modified parasternal short axis view displaying anterocephalic malalignment of the VSD (arrow) causing right ventricular outflow tract obstruction. (C) Computed tomography scan presenting abnormal right lower pulmonary vein (arrow) draining into the inferior vena cava, the pathognomonic “scimitar sign”. (D) Transcatheter angiography image of the sequestrating artery from the descending aorta. Ao=aorta; LV=left ventricle; RV=right ventricle; RVOT=right ventricular outflow tract

A seven-month-old infant, weighing 5.8 kg, was referred to our outpatient clinic for further evaluation of a pathological murmur located at the left second intercostal border with a 2/6 systolic pattern. The oxygen saturation in room air was 86% and the infant was otherwise healthy. Electrocardiogram showed normal sinus rhythm with 120 beats per minute, right axis deviation (150°) and a positive T wave in V_1_, suggestive of right ventricular hypertrophy. Echocardiography revealed the diagnosis of TOF with hypoplastic pulmonary arteries, right aortic arch, persistent left superior vena cava and patent foramen ovale. In addition, an unexpected turbulent flow in the inferior vena cava raised the suspicion of partial abnormal pulmonary venous return. Cardiac computed tomography, which was used to clarify the anatomical connections, enlightened a partially abnormal venous drainage from the right lower pulmonary vein to the inferior vena cava and an anomalous arterial blood supply from the descending aorta to the sequestrating pulmonary segment, in addition to the abovementioned echocardiographic findings (Figure 1). Cardiac catheterization was performed; during the angiographic levophase of the right lower pulmonary vein, partial anomalous venous drainage into the inferior vena cava was noted, in addition to diffuse hypoplasia of the right pulmonary artery and a systemic arterial supply from the abdominal aorta. In the light of these findings, the diagnosis of scimitar syndrome accompanying TOF was obtained. In the same session, the arterial supply for sequestration was occluded by an 8 mm Amplatzer^TM^ vascular plug 4 device (AGA Medical Corporation, Plymouth, MN). The patient was operated on the same day under cardiopulmonary bypass. The ventricular septal defect closure was performed, a transannular patch was placed for reconstruction of the right ventricular outflow tract, and the lower pulmonary vein was directed to the left atrium via an intra-atrial tunnel. The patient was weaned uneventfully from cardiopulmonary bypass, recovered well after surgery with normal saturation and was discharged home after 10 days. Long-term clinical follow-up of the patient is free of complications so far.

## DISCUSSION

This case report brings awareness to the abnormal pulmonary venous connections that may rarely coexist in patients with TOF. A large series with 1,183 TOF cases, reported by Redington et al.^[^^1^^]^, underlines this uncommon association by reporting only 7 cases with abnormal pulmonary venous return (0.6%), in which solely one patient had scimitar syndrome. As patients with TOF are usually cyanotic, abnormal pulmonary venous connections are infrequently suspected. In our case, the patient was diagnosed with scimitar syndrome, after the suspicion of an abnormal flow connecting to the inferior vena cava on echocardiography, together with a radiological demonstration of right lung hypoplasia, disoriented right lower pulmonary vein, sequestrating artery originating from the descending aorta. Cross-sectional echocardiography should be performed precisely to track all pulmonary venous returns and additional intracardiac anomalies, but cardiac catheterization or computed tomography must be considered if normal pulmonary venous drainage cannot be clearly shown by non-invasive techniques. In the light of our case report and the current literature, the detection of an abnormal flow connection to the inferior vena cava on echocardiography may raise the suspicion that scimitar syndrome lies underneath in those patients with TOF^[^^1^^,^^4^^-^^6^^]^.

Although surgery allows complete correction, transcatheter occlusion of the anomalous systemic arterial supply has been reported as an alternative and less invasive method to surgical ligation in symptomatic patients with scimitar syndrome. Moreover, transcatheter occlusion of aberrant vessels to the sequestered pulmonary parts has been reported beneficial for the reduction of recurrent respiratory infections^[^^7^^,^^8^^]^. Therefore, in our patient we decided to use the hybrid combination of initial transcatheter occlusion of the arterial supply to the sequestration by a vascular plug, and corrective surgery for both TOF and disoriented pulmonary vein shortly after.

## CONCLUSION

In conclusion, the rare association of TOF with scimitar syndrome should be kept in mind. In case of suspicion, echocardiographic evaluation should be preferred first since it is non-invasive and easily applicable. For the evaluation of especially extracardiac structures, computed tomography or conventional angiography should be considered thereafter. Hybrid combination of initial transcatheter occlusion and corrective surgery could be a good option in patients with TOF and scimitar syndrome.

**Table:** 

Authors' roles & responsibilities	
BÇ	Substantial contributions to the conception or design of the work; or the acquisition, analysis, or interpretation of data for the work; agreement to be accountable for all aspects of the work in ensuring that questions related to the integrity of any part of the work are appropriately investigated and resolved; final approval of the version to be published
SUA	Substantial contributions to the conception and design of the work; interpretation of data for the work; drafting the work, revising it critically for important intellectual content; final approval of the version to be published
AG	Drafting the work, revising it critically for important intellectual content; final approval of the version to be published
